# Singly Flagellated *Pseudomonas aeruginosa* Chemotaxes Efficiently by Unbiased Motor Regulation

**DOI:** 10.1128/mBio.00013-16

**Published:** 2016-04-05

**Authors:** Qiuxian Cai, Zhaojun Li, Qi Ouyang, Chunxiong Luo, Vernita D. Gordon

**Affiliations:** a Center for Quantitative Biology, Academy for Advanced Interdisciplinary Studies, Peking University, Beijing, China; b Department of Physics, Center for Nonlinear Dynamics, University of Texas at Austin, Austin, Texas, USA; c Center for Microfluidic and Nanotechnology, The State Key Laboratory for Artificial Microstructures and Mesoscopic Physics, School of Physics, Peking University, Beijing, China

## Abstract

*Pseudomonas aeruginosa* is an opportunistic human pathogen that has long been known to chemotax. More recently, it has been established that chemotaxis is an important factor in the ability of *P. aeruginosa* to make biofilms. Genes that allow *P. aeruginosa* to chemotax are homologous with genes in the paradigmatic model organism for chemotaxis, *Escherichia coli*. However, *P. aeruginosa* is singly flagellated and *E. coli* has multiple flagella. Therefore, the regulation of counterclockwise/clockwise flagellar motor bias that allows *E. coli* to efficiently chemotax by runs and tumbles would lead to inefficient chemotaxis by *P. aeruginosa*, as half of a randomly oriented population would respond to a chemoattractant gradient in the wrong sense. How *P. aeruginosa* regulates flagellar rotation to achieve chemotaxis is not known. Here, we analyze the swimming trajectories of single cells in microfluidic channels and the rotations of cells tethered by their flagella to the surface of a variable-environment flow cell. We show that *P. aeruginosa* chemotaxes by symmetrically increasing the durations of both counterclockwise and clockwise flagellar rotations when swimming up the chemoattractant gradient and symmetrically decreasing rotation durations when swimming down the chemoattractant gradient. Unlike the case for *E. coli*, the counterclockwise/clockwise bias stays constant for *P. aeruginosa*. We describe *P. aeruginosa*’s chemotaxis using an analytical model for symmetric motor regulation. We use this model to do simulations that show that, given *P. aeruginosa*’s physiological constraints on motility, its distinct, symmetric regulation of motor switching optimizes chemotaxis.

## IMPORTANCE

Chemotaxis has long been known to strongly affect biofilm formation by the opportunistic human pathogen *P. aeruginosa*, whose essential chemotaxis genes have homologues in *E. coli*, which achieves chemotaxis by biasing the relative probability of counterclockwise and clockwise flagellar rotation. However, the physiological difference between multiflagellated *E. coli* and singly flagellated *P. aeruginosa* implies that biased motor regulation should prevent *P. aeruginosa* populations from chemotaxing efficiently. Here, we used experiments, analytical modeling, and simulations to demonstrate that *P. aeruginosa* uses unbiased, symmetric regulation of the flagellar motor to maximize its chemotaxis efficiency. This mode of chemotaxis was not previously known and demonstrates a new variant of a paradigmatic signaling system in an important human pathogen.

## INTRODUCTION

Bacterial chemotaxis allows a fast response to changing environments and is one of the most-studied signal transduction systems in biology. By far the best-understood model organism for chemotaxis is *Escherichia coli*. On a cellular level, *E. coli* alternates times of straight swimming, known as “runs,” with times of directional change, known as “tumbles” (1). *E. coli* has an increased probability of running when it is moving toward a higher concentration of chemoattractant and an increased probability of tumbling when it is moving toward a lower concentration of chemoattractant (1). Biasing the probability of a run or tumble is accomplished by the chemotaxis signal transduction network (2): chemoreceptors sense changes in the environment and transmit this information to the downstream regulator protein CheY-phosphate (CheY-p); binding of CheY-p to a flagellar motor increases the likelihood that it will rotate clockwise (CW) (3, 4). *E. coli* has multiple helical flagella that bundle when they are rotating counterclockwise (CCW), resulting in a run, and debundle when they are rotating CW, resulting in a tumble. Thus, for multiflagellated *E. coli*, controlling the probability of flagella rotating CCW or CW controls the likelihood of continuing in the same direction or changing direction.

The genes controlling chemotaxis in *E. coli* are homologous to the genes controlling chemotaxis in many other bacterial organisms, including *Pseudomonas aeruginosa*, *Rhodobacter sphaeroides*, and *Bacillus subtilis* (5). For *P. aeruginosa* specifically, chemotaxis is also known to be important for the formation of multicellular, pathogenic biofilms (6–8). However, unlike *E. coli*, *P. aeruginosa* has only a single polar flagellum. Therefore, CW flagellar rotation will result in *P. aeruginosa* being pulled backwards in a straight trajectory (9), not tumbling as for *E. coli*. Another singly flagellated bacterial species, *Vibrio alginolyticus*, explores space using a flagellar “flick” that can sample a broad range of angles and by changing the relative duration of “forward” runs (CW flagellar rotation) and “backwards” runs (CCW flagellar rotations) (10). A recent study on tethered *P. aeruginosa* revealed a run-reverse-turn motility pattern (11). However, how *P. aeruginosa* regulates motility to achieve chemotaxis in a heterogeneous environment is not well understood. This is a gap in our understanding of a fundamental process in biological signaling and adaptation for an important human pathogen.

Here, we used microfluidics to control the nutrient environment and quantitative analysis of microscopic movies to determine the cell-level mechanism of chemotaxis for *P. aeruginosa*. We found that *P. aeruginosa* efficiently chemotaxed, despite very limited angular changes in orientation, by decreasing the likelihood of changing direction when it was going up a favorable gradient and increasing the likelihood of changing direction when it was going down the same gradient. Unlike the case for *E. coli*, we found that *P. aeruginosa* regulated the length of time between switches in rotation direction but left the relative probability of CW and CCW flagellar rotation unchanged. This symmetric motor regulation indicates that the flagellum can push or pull the *P. aeruginosa* cell toward the attractant. We used our experimental results to develop an analytical model for *P. aeruginosa* chemotaxis. We varied the parameters characterizing the probability of the flagellar motor switching rotation direction, and we found that a symmetric regulation of CCW and CW rotations resulted in stronger, more precise chemotactic response than did asymmetric regulation. Our results demonstrate another way of achieving chemotaxis, by adjustment of the flagellar motor switching complex, and imply that even though the switching complex protein is conserved across bacterial species, it likely has evolved variations to be compatible with different types of swimming motility.

## RESULTS AND DISCUSSION

### *P. aeruginosa* swims in time-reversible, back-and-forth trajectories with little change in direction

**FIG 1:**
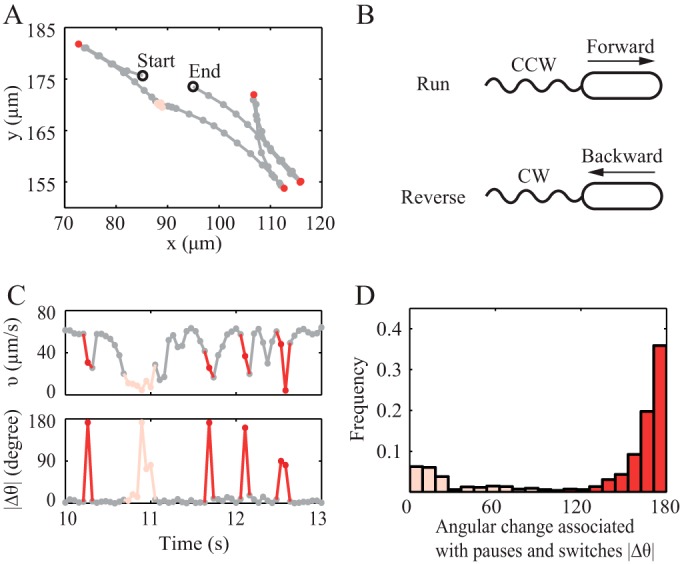
*P. aeruginosa* swimming behavior in chemotaxis buffer. (A) A representative trajectory with forward and backward runs (grey), a pause (pink), and switches in direction (red). The time interval between two consecutive circles is 0.053 s. (B) Schematic diagram of forward and backward swimming motility. (C) The corresponding instantaneous speed and angular change per 0.053 s of the trajectory in panel A. (D) The probability distribution of angular change associated with pause and switch. Red, switch; pink, pause.

To assess the directional modulations available to *P. aeruginosa*, we characterized the trajectories of wild-type *P. aeruginosa* strain PAO1 swimming in attractant-free microfluidic channels (see Materials and Methods; see also Fig. S1A in the supplemental material). To avoid the effects of hydrodynamic interactions with the walls (12), our microscope was focused in the middle of the chamber, ~65 µm away from the top and bottom surfaces. Unlike the 3-step “run-reverse-flick” motility in monotrichous *V. alginolyticus* (10), *P. aeruginosa* takes a 3-step swimming pattern of run-reverse-pause (Fig. 1A and B), similar to previous findings for *Pseudomonas putida* (13) and *P. aeruginosa* in tethering experiments (11). A “pause” is a sudden decrease in the speed to nearly 0 µm/s (Fig. 1C); the swimming direction after such a pause tends to remain the same (Fig. 1D, lower peak). A reverse is associated with an ~180° change in swimming direction (Fig. 1C and D, higher peak). The cell body itself is not found to undergo tumbling or a significant change in orientation when imaged at 19 frames/s. Therefore, we classified changes in velocity as resulting from motor reversing (angle changes of more than 90°) (Fig. 1, red) or from motor pausing (angle changes of less than 90°) (Fig. 1, pink). About 24% of velocity changes fell into the “pausing” category, with angular changes of 27 ± 25°. The other 76% of the velocity changes were associated with a switch in swimming direction, with average angular changes of 166 ± 15°. Of all the trajectories observed, pausing took up only 4.3% of the total tracking time and switching direction took up 8.8%. This percentage was not affected by the presence of gradients (see Table S1 in the supplemental material). The distribution of angular changes and durations of pauses and switches were also unaffected by the presence of the gradient (see Fig. S2 and Table S1).

A previous study suggests that pausing can help bacteria change their angular orientation by rotational diffusion (11). To assess the importance of this effect in our system, we calculated the rotational diffusion coefficients for trajectories without pausing and with pausing events (see Fig. S3 in the supplemental material). The rotational diffusion coefficient was 0.026 rad^2^/s for nonswitching trajectories without pauses and only 0.038 rad^2^/s for nonswitching trajectories with pauses. The latter is only 67% of the rotational diffusion coefficient of 0.057 rad^2^/s for *E. coli* (14). From these results, we conclude that *P. aeruginosa* is very limited in its capacity to modulate swimming direction, because it has neither high rotational diffusion nor active directional randomization.

**FIG 2:**
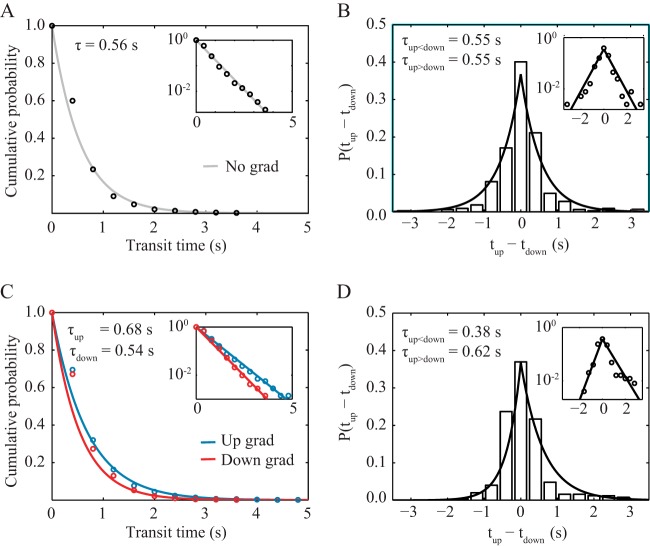
Transit time statistics for cells swimming up and down microfluidic channels. (A) A cumulative probability distribution of transit times under an attractant-free (control) condition shows that all observed transits are described by one distribution. (C) In contrast, in a 0.225 µM/µm linear gradient of serine, transit times up the gradient are described by a different distribution than are transit times down the gradient, with the characteristic time up the gradient being greater than both the characteristic time down the gradient and the characteristic time with no gradient. Another measure of directional bias is given by the probability distribution of the difference in consecutive run times up and down the channel (*t*_up_ − *t*_down_). Blue, up gradient; red, down gradient. (B) Under attractant-free (control) conditions, this distribution is symmetric about 0, indicating no directional bias. The sample size is 322 pairs of up and down trajectories. (D) In a 0.225 µM/µm linear gradient of serine, the distribution is asymmetric and biased toward positive values. The sample size is 339 pairs of up and down trajectories. The insets show the same data plotted in semilog form and fitted to linear functions, as follows: ln *P* = −*t*/τ in panels A and C and either ln *P* = *t*/τ_up<down_ + *b* for (*t*_up_ − *t*_down_ < 0) or ln *P* = −*t*/τ_up>down_ + *b* for (*t*_up_ − *t*_down_ > 0) in panels B and D.

We also measured the transit times in the attractant-free channel, where we defined transit time as the elapsed time between two consecutive switches in direction. We found that the transit times followed an exponential distribution, *P*(*X* > *t*) = *e*^–*t*/τ^, with a characteristic transit time τ of 0.56 s (Fig. 2A). Moreover, the distribution of consecutively measured transit times was symmetric about zero (Fig. 2B), indicating that, on average, cells spent equal amounts of time swimming up the channel and down the channel. These findings demonstrate that *P. aeruginosa*’s swimming motility has no intrinsic bias in direction in the absence of a chemoattractant gradient.

### *P. aeruginosa* chemotaxes by modulating swimming times up and down an attractant gradient.

To elucidate how *P. aeruginosa* modulates this bidirectional, symmetric swimming pattern to achieve chemotaxis, we applied a linear gradient of the chemoattractant serine in the microfluidic channel such that the average concentration of serine in the field of view was 140 µM and the gradient was 0.225 µM/µm. Unlike the case for the attractant-free channel, the trajectories when swimming up the gradient became longer on average, with a characteristic transit time of 0.68 s, and trajectories when swimming down the gradient became shorter on average, with a characteristic transit time of 0.54 s (Fig. 2C). Furthermore, the difference in consecutive transit times showed a bias toward positive values (Fig. 2D). These results indicate that *P. aeruginosa* bacteria spent longer times traveling up the serine gradient than traveling down, which is consistent with achieving chemotaxis toward serine. We also note that our measurements unavoidably undercounted the probability of longer transit durations, since trajectories that did not undergo at least two switches in direction before they ran out of the 200-µm by 200-µm field of view were not included. Thus, the actual shift in characteristic run times imposed by the presence of the gradient is likely greater than the shift we were able to measure.

### CW/CCW bias is largely unaffected by a chemoattractant gradient.

*E. coli* differentiates up-gradient and down-gradient transit times by modulating the bias for CCW versus CW flagellar rotation. To elucidate how a chemoattractant gradient affects CCW and CW flagellar rotation for *P. aeruginosa*, we tethered cells by their flagella onto the bottom coverslip of a flow cell. The rotation of a cell thus reflected the rotation of its flagellum (15). In the absence of chemoattractant, we measured equal average angular speeds for CW and CCW rotations (see Table S2 in the supplemental material), and the CCW and CW durations were 1.36 ± 1.53 s and 1.17 ± 1.22 s, respectively. These findings for tethered cells are consistent with our finding that swimming *P. aeruginosa* bacteria had the same speed for both forward and backward runs (see Fig. S4 in the supplemental material) and indicate that there is little intrinsic CW/CCW bias.

**FIG 3:**
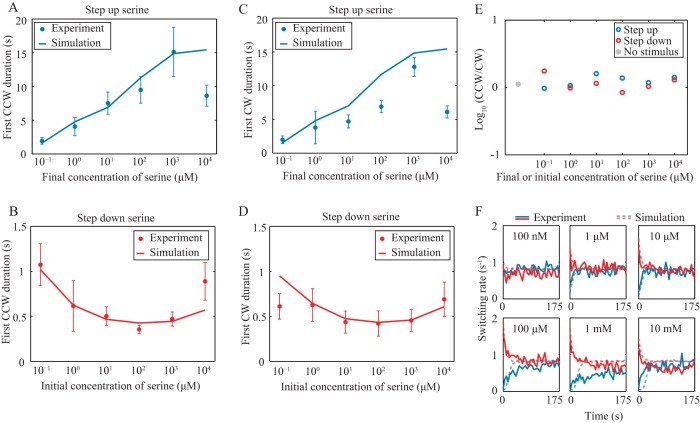
CCW and CW durations when step function concentrations of serine are imposed on tethered cells. (A and C) First CCW and CW durations for stepped-up stimuli. (B and D) First CCW and CW durations for stepped-down stimuli. Error bars represent the standard errors of the means. (E) The ratios of the durations of CCW and CW rotation intervals for the same data shown in panels A to D. (F) Adaptation of switching rates after stimulus. Blue and red represent stepped-up and stepped-down stimulus, respectively. CCW and CW designate the direction of flagellar rotation.

By controlling the flow of serine-free and serine-containing medium, we imposed stepped-up and stepped-down changes in serine concentration (see Materials and Methods; see also Fig. S5 in the supplemental material). A step up in concentration allowed us to probe the behavior of bacteria when swimming up a gradient, and a step down in concentration allowed us to probe the behavior of bacteria when swimming down a gradient. When the serine concentration was stepped up, we found that the duration of rotation intervals increased—i.e., the likelihood of switching direction decreased (Fig. 3A and C). Moreover, we found that the rotation duration immediately after the serine stimulus was applied had a monotonically increasing relationship to the magnitude of the increase in serine concentration for the range from 100 nM to 1 mM. This indicates that if a bacterium is swimming up an attractant gradient, the likelihood of switching direction to go the “wrong” way down the gradient is a monotonically decreasing function of the gradient’s steepness. When the serine concentration was stepped down, the average rotation duration decreased (Fig. 3B and D), and this decrease was monotonic with the initial serine concentration for the range from 100 nM to 100 µM. This indicates that if a bacterium is swimming down an attractant gradient, the likelihood of switching direction to go the “right” way up the gradient is a monotonically increasing function of the gradient’s steepness. The time that the bacterium needed to recover to its original state also increased with the gradient steepness for the range from 100 nM to 1 mM, with the adaptation time ranging from 5 to 100 s (Fig. 3F).

The ratio of the average CW duration to the average CCW duration is close to unity for all stimuli (Fig. 3E), which indicates that the CW/CCW bias is largely unaffected by the gradient. This is strikingly unlike the case for *E. coli*, for which the initial response to an attractive stimulus is exclusively in the form of CCW rotation (4, 16, 17). In contrast, our results shown in Fig. 3 show that if a cell was swimming along a linear gradient, it biased its motility by increasing the time travelling up gradient (τ_+_) and decreasing the time travelling down gradient (τ_−_), no matter whether the motion was a forward or a backward run (Fig. 1B). As a result, a net drift velocity *v*(τ_+_ − τ_−_)/(τ_+_ + τ_−_) would be expected.

### A two-state model with symmetric motor regulation describes *P. aeruginosa* chemotaxis.

The basic chemotaxis signal pathways are conserved among different bacterial species (5, 18), and the general schema is summarized in Fig. S6 in the supplemental material. In brief, the binding of a ligand to the transmembrane chemoreceptor causes a conformational change in the receptor which reduces the activity of CheA. Activated CheA phosphorylates CheY, which regulates the direction of flagellum rotation. CheR and CheB methylate and demethylate the chemoreceptor to allow it to adapt its response to different chemoattractant concentrations and gradients. To better understand *P. aeruginosa*’s chemotaxis behavior, we propose a single-cell model that is modified from the coarse-grained signaling pathway-based *E. coli* chemotaxis simulator (SPECS) (19).

Specifically, in *P. aeruginosa*, there are five clusters of chemotaxis-like genes. Clusters I, II, and V are involved in swimming-related chemotaxis. The genes *cheA*, *cheW*, *cheY*, *cheZ*, *cheB*, and *cheR*, from clusters I and V, are essential for *P. aeruginosa* to chemotax (20, 21) and have homologous counterparts in *E. coli*. Similarly to *E. coli* chemoreceptors, the chemoreceptors in *P. aeruginosa* form complexes and assemble into localized clusters (22); clustering can promote cooperativity between chemoreceptors. However, the *E. coli* and *P. aeruginosa* chemoreceptors are structurally different in the ligand-binding region, which causes the two species to have different recognition and response profiles (23, 24). The ligand serine, which we used as a chemoattractant, can bind to the *P. aeruginosa* chemoreceptor PctA (23–25). The signal output of PctA as monitored by the fluorescence resonance energy transfer (FRET) assay shows a high response sensitivity for various amino acid concentrations spanning three orders of magnitude (26). Our results on the behavior level also confirm that *P. aeruginosa* has a wide dose-response range (Fig. 3). Chemoreceptor clustering and high sensitivity over a wide range of stimuli both indicate that there is likely cooperativity among *P. aeruginosa* chemoreceptors. Cooperativity is known to be crucial for *E. coli* chemotaxis by controlling sensitivity (27, 28) and the signal amplification (2). Cooperativity allows the average activity of chemoreceptors to be described by the two-state Monod-Wyman-Changeux (MWC) model (19, 29), as follows:

(1) $$a=\frac{1}{1+\exp\left\{N\varepsilon \left[m,\left(L\right)\right]\right\}}$$

where *N* is the number of cooperative receptors in the cluster. “*m*” is the methylation level of the receptor, and “(*L*)” is the ligand concentration which is a function of space and time. ε is the free energy difference between the active state and the inactive state of the receptor, as follows:

(2) $$\varepsilon \left[m,\left(L\right)\right]=f_{m}-\ln\frac{1+\left(L\right)/K_{A}}{1+\left(L\right)/K_{I}}$$

where *K*_*I*_ and *K*_*A*_ are the dissociation constants of the ligand to the inactive and the active receptor. *f_m_* is the free energy difference due to the methylation and demethylation of the chemoreceptor and is set to be linear in *m*:

(3) $$f_{m}=\alpha \left(m_{0}-m\right)$$

Methylation and demethylation are the two enzymatic reaction processes catalyzed by CheR and CheB. Assuming that CheR only catalyzes the inactive receptors and CheB only catalyzes the active ones, the methylation rate is described as follows:

(4) $$\frac{dm}{dt}=V_{R}\left(a\right)\frac{1-a}{K_{R}+1-a}-V_{B}\left(a\right)\frac{a}{K_{B}+a}$$

where *K_R_* and *K_B_* are the Michaelis constants for methylation and demethylation, respectively. *V_R_*(*a*) and *V_B_*(*a*) are the maximum catalytic rates when CheR and CheB are saturated with substrates. A recent study indicates that *V_R_*(*a*) and *V_B_*(*a*) are not constants with the activity of CheA (30). There is a sharp increase in *V_R_*(*a*) and *V_B_*(*a*) when the activity of receptors is far from steady state. Thus, we use a piecewise linear form for both *V_R_*(*a*) and *V_B_*(*a*), as follows: $V_{R}\left(a\right)=V_{R}(0)[1+\theta \left(a_{R}-a\right)\frac{a_{R}-a}{a_{R}}r_{R}],\;V_{B}\left(a\right)=V_{B}(0)[1+\theta \left(a-a_{B}\right)\frac{a-a_{B}}{1-a_{B}}r_{B}].$ θ(*x*) is the unit step function such that [θ(*x*) = 1 for *x* > 0, θ(*x*) = 0 otherwise]. For simplicity, we let *K_R_* = *K_B_*, *V_R_*(0) = *V_B_*(0), and *r_R_* = *r_B_*.

**FIG 4:**
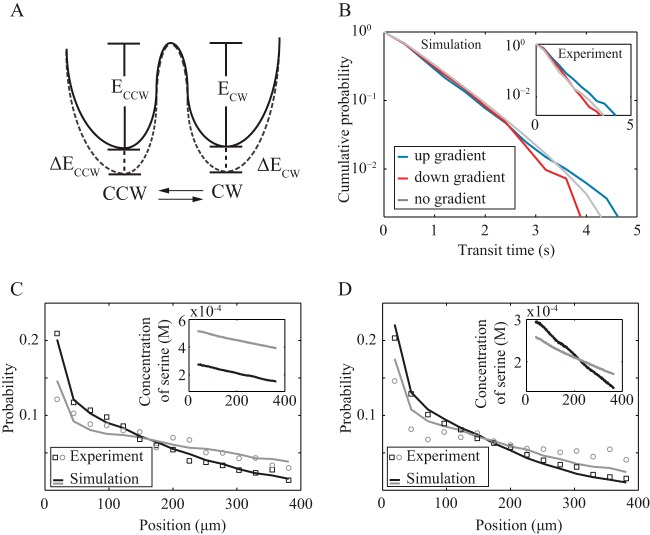
Model descriptions and predictions. (A) A possible model for motor switching. Binding of CheY-p changes the free energies of CCW and CW (solid line to dashed line) by Δ*E*_CCW_ and Δ*E*_CW_. This assumption gives our model as described in the text, which we use to obtain the following results. (B) From simulations, the probability distribution of run times up and down the chemoattractant gradient. The inset shows the experiment data from Fig. 2A and C. Also from simulations, we predict the cell density distributions in a 600-µm by 200-µm microfluidic channel with equal serine gradients but different average serine concentrations (C) and different serine gradients but the same average serine concentrations (D). Lines are from the model simulation. Hollow circles and squares are experiment data, which were not used in fitting the model parameters but are well described by simulation. Insets are the serine concentration profiles across the channel, color coded to match the corresponding cell density profiles.

For motor switching regulation, our experiments clearly show that CheY must regulate the rotation of the flagellum motor differently in *P. aeruginosa* than in *E. coli*. We assume that the energy landscape of these two states is affected by the binding of CheY-p and, therefore, by CheA activity and use a thermal isomerization model (Fig. 4A) to estimate the behavior of the motor free energies (31), as follows:

(5) $$E_{\text{CCW}}=k_{1}k_{B}T-\beta_{1}\frac{a}{a+\gamma }k_{B}T$$

(6) $$E_{\text{CW}}=k_{2}k_{B}T-\beta_{2}\frac{a}{a+\gamma }k_{B}T$$

In equations 5 and 6, *k*_1_*k_B_T* and *k*_2_*k_B_T* are the free-energy barriers to switching the rotation direction in the absence of any CheY-p binding to the motor. The second term is the free energy change induced by CheY-p binding. $\frac{a}{a+\gamma }$describes the motor occupancy by CheY-p. γ is the CheA activity when the motor is half occupied by CheY-p. The free energies are affected by this occupancy by a factor of β*k_B_T*. The rates at which the flagellar motor switches from CCW to CW and from CW to CCW are as follows:

(7) $$P_{\text{CCW}\to \text{CW}}=A\exp\left(-\frac{E_{\text{CCW}}}{k_{B}T}\right)$$

(8) $$P_{\text{CW}\to \text{CCW}}=A\exp\left(-\frac{E_{\text{CW}}}{k_{B}T}\right)$$

following a Boltzmann distribution.

From equations 5 to 8, we have $\ln\left(\frac{P_{\text{CW}\to \text{CCW}}}{P_{\text{CCW}\to \text{CW}}}\right)=(k_{1}-k_{2})-(\beta_{1}-\beta_{2})\frac{a}{a+\gamma }$. The results of our tethering experiments show that the ratios of CCW and CW durations are nearly constant; hence, (*P*_CW→CCW_/*P*_CCW→CW_)~1 across a wide range of applied stimuli, and we make the simplifying approximations β_1_ − β_2_ and *k*_1_ − *k*_2_. This corresponds to switching from CCW to CW and from CW to CCW being almost equally probable. We also incorporate pausing events in our simulation as an increased rotational diffusion. The rotational diffusion coefficient is 0.038 rad^2^/s, as mentioned above. We fit this model to data from our tethering experiments to get values for ln *A* − *k*_1_, ln *A* − *k*_2_, β_1_, β_2_, and γ (see Table S3 in the supplemental material).

To validate the model, we simulated the transit times up and down gradients, as well as the resulting cell density profiles (Fig. 4B). We found that for short transit times, such those where the bounding reversals happened within a second of each other, the probabilities of swimming up or down the gradient were comparable. However, for long transit times, the probabilities of swimming up or down the gradient became well resolved and swimming up the gradient was more likely. These simulation results agree well with the results of our microfluidic experiments (Fig. 4B, inset). Long transit events will impact the mass transport of the bacterial population more than will short transits. Indeed, we find that our simulated cell density profiles agree quantitatively with the experimentally measured density distributions of *P. aeruginosa* in different linear gradients (Fig. 4C and D).

### Symmetric motor regulation provides efficient chemotaxis for *P. aeruginosa*.

**FIG 5:**
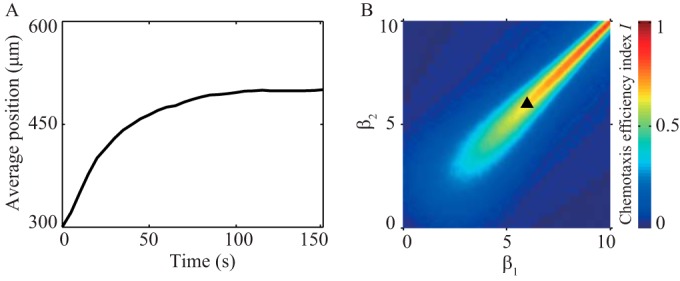
Localization of a bacterial population in response to a chemotactic gradient. (A) Using model parameters obtained by fitting to experiment data, we simulate the dynamics of localization in a closed, square chamber with a 0.75 µM/µm linear gradient. (B) The chemotaxis efficiency index *I* for different combinations of β_1_ and β_2_. The triangle is the result simulated using experimentally derived parameters for our *P. aeruginosa* chemotaxis model.

Our model for *P. aeruginosa* chemotaxis has two key differences from the corresponding model for *E. coli* chemotaxis (19). First, *P. aeruginosa* regulates CCW and CW rotations symmetrically, whereas *E. coli* regulates CCW and CW rotations asymmetrically, to change the relative probability of rotating CCW or CW. Second, *P. aeruginosa* uses run-reverse to change its direction of motion, rather than run-tumble as for *E. coli*. To determine whether symmetric regulation of motor direction achieves better chemotaxis for *P. aeruginosa* than would *E. coli*’s asymmetric, CCW/CW-biasing regulation, we simulated *P. aeruginosa*’s chemotaxis in a linear gradient in a closed square chamber (600 µm by 600 µm). We varied β_1_ and β_2_ over the range from 0 to 10. In our simulations, the cells started from a uniform distribution and gradually moved toward the end with a higher concentration of attractant. The average position of the entire population across the channel reached a steady state in ~120 s for the parameters determined by fitting the model to experiment data (Fig. 5A). We used the index *I* = [mean(*y_i_*) − y_c_]/*y_c_* to measure chemotaxis efficiency, where *y_c_* is the *y* coordinate at the center of the channel and mean(*y_i_*) is the average *y* coordinate of the bacterial population at steady state. Thus, *I* = 1 corresponds to maximum efficiency and *I* = 0 corresponds to minimal efficiency. For the 10-fold range of values we sampled for β_1_ and β_2_, our simulations show that the best efficiency is achieved when β_1_ = β_2_ (Fig. 5B). Small departures from this line of equivalence give rise to large decreases in *I*. This indicates that singly flagellated organisms, such as *P. aeruginosa*, that use symmetric motor regulation can chemotax more efficiently than if they used unbalanced motor regulation.

In our quantitative study of *P. aeruginosa* chemotaxis on the single-cell and population levels, we found that *P. aeruginosa* adjusts the rate at which the rotating flagellum switches directions, while keeping the CW/CCW bias roughly constant. As a result, *P. aeruginosa* can be either pushed or pulled toward chemoattractants, regardless of which way the cell head faces (Fig. 1B). We have shown that an analytical model incorporating this symmetric approach to motor regulation fits our single-cell data well and can quantitatively predict the population-level migration we measured experimentally. For singly flagellated bacteria like *P. aeruginosa*, symmetric regulation of the flagellar motor gives more efficient chemotaxis than does the biased motor regulation used by multiflagellated *E. coli*.

Although it is certain that genes for chemotaxis are conserved across species, chemotaxis pathways in some species are far more complex and less understood than that in *E. coli* (5, 18). Our results imply that the protein sequences and structures for the motor switching complex and/or the regulation of other molecules in the chemotaxis signal-transduction pathway are likely different among species. Moreover, chemotaxis gene cluster II in *P. aeruginosa*, in addition to clusters I and IV, comprising a complete set of *che* genes, is also involved in chemotaxis (32). In future work, it would be interesting to know to what degree these clusters interact in chemotaxis signaling.

## MATERIALS AND METHODS

### Cell strains, culture media, and cell preparation.

We used the Nottingham wild-type (WT) PAO1 strain of *P. aeruginosa*. Bacteria were streaked from frozen stock onto lysogeny broth (LB) agar. Colonies were inoculated into minimal salts (MS) medium (7.0 g of K_2_HPO_4_, 3.0 g of KH_2_PO_4_, 1.5 g of KNO_3_, 0.05 g of MgSO_4_·7H_2_O, and 2.5 mg of FeCl_3_·6H_2_O) containing 0.4% (wt/vol) sodium succinate and incubated, shaking, overnight at 37°C (33, 34).

The overnight cultures were diluted 100× and then subcultured in fresh MS medium for about 6 h, at which point cells were in exponential phase with an optical density at 600 nm of 0.1 to 0.15 (Spectronic). The cultures were then centrifuged at 300 × *g* for 4 min and resuspended in a modified chemotaxis buffer (CB), consisting of 5 mM Mg^2+^, 15 µM EDTA, and 10 mM dl-lactic acid in phosphate-buffered saline (PBS), pH 7.0 (33–35).

### Microdevice preparation and operation and fluorescent calibration.

A microfluidic device modified from a previous design was fabricated on a silicon wafer by standard two-layer lithography (see Fig. S1A in the supplemental material) (36). The first, lower layer consists of 8 to 12 parallel channels. The second, upper layer contains two large wells with a diameter of 5 mm and an observation channel. The observation channel is connected directly to one of the large wells (the sink well) and indirectly to the other large well (the source well), via smaller, agarose-filled channels in the lower layer. The agarose gel ensures that all transport between the source well and the observation chamber is diffusive.

For our experiments to measure the run times of trajectories (see Fig. S1B in the supplemental material), a bacterium-containing channel with a size of 2,000 µm by 400 µm by 130 µm (length [L] by width [W] by height [H]) was connected with 12 agarose-filled channels with a size of 800 µm by 20 µm by 25 µm (L by W by H). For our measurements of cell density profiles (see Fig. S1C), a bacterium-containing channel with a size of 600 µm by 200 µm by 130 µm (L by W by H) was connected with 8 agarose-filled channels with a size of 300 µm by 15 µm by 25 µm (L by W by H).

This device was cast in polydimethylsiloxane (PDMS) (RTV 615 with curing agents; Momentive). The two wells were punched with a biopsy punch (5 mm in diameter, World Precision Instruments). The resulting PDMS device was cleaned using oxygen plasma (March Plasma CS170IF reactive ion etcher [RIE] etching system) and then bound to a clean glass coverslip. While the surface of the PDMS was still hydrophilic, we filled the parallel, source-to-observation connecting channels with 2% to 3% low-melting-point agarose (Sigma-Aldrich) at 60°C; the agarose was fluid at this temperature and solidified with cooling to room temperature. Sixty microliters of water was added to the wells. Prepared chips were stored in the refrigerator overnight at 4°C. Water vapor permeated the whole observation channel.

To calibrate the relationship between the concentration of a chemoattractant in the source well and the concentration profile in the observation chamber, 60 µl of serine solution (pH 7.0) with 20 µM fluorescein (Sigma-Aldrich) was added to the source well. In the sink well, we added 60 µl CB. We determined the resulting gradient by measuring the fluorescence along the observation chamber, using fluorescence microscopy. As a control, the fluorescence intensity was normalized to the signal obtained when the fluorescein was added in both sink and source well. All the channels were stored at room temperature in wet, dark environments for at least 8 h before microscopy.

### Imaging swimming trajectories and cell locations in the microfluidic chemotaxis device.

Before microscopy imaging, we replaced the CB in the sink well with 10^4^/ml *P. aeruginosa* cells in CB. An Olympus IX71 inverted microscope and 10× phase-contrast objective were used for measuring cell trajectories and locations. Images were taken at 19 frames per second by a QImaging EXi blue charge-coupled device (CCD) camera controlled using QCapture Pro 6 imaging software. For measurements of swimming trajectories (see Fig. S1B in the supplemental material), we took images in the region with an average serine concentration of 140 µM and a gradient of 0.225 µM/µm. For measuring the location of the bacterial population (see Fig. S1C), we used agarose gel to block the connection of the channel to the bacterium-containing sink chamber so that the number of bacteria in the observation channel was fixed. The chamber and all solutions were maintained at 30°C using an incubator enclosure (Precision Plastics) surrounding the microscope stage area. To profile the density distribution of the bacterial population, the normalized probability of cells was averaged over 300 images.

### Analysis of the trajectories of swimming cells.

The centroids of cells were detected and traced by particle-tracking codes written in MatLab (37). Trajectories with an average speed of less than 15 µm/s or with a tracked duration of less than 3 s were discarded. Trajectories with helix or pseudohelix shapes (38) were only ~5% of the total trajectories and were also excluded from analysis. The statistics in the text, if not specifically mentioned, are mean results ± standard deviations (SD). Changes in swimming velocity were identified as an abrupt decrease in instantaneous speed or a high angular change, as described previously (13).

In brief, to identify abrupt decreases in speed, we first identified each local minimum in instantaneous speed, defined to occur at time *t*_min_, and the local maxima immediately before and after *t*_min_, at times *t*_1_ and *t*_2_. If the relative change of velocity was Δ*v*/*v*(*t*_min_) > 6, where Δ*v* =& max[*v*(*t*_1_) − *v*(*t*_min_), *v*(*t*_2_) − *v*(*t*_min_)], we considered the trajectory to be undergoing angular change during the time interval around *t*_min_ such that *v*(*t*) < *v*(*t*_min_) + 0.2Δ*v*. To identify large angular changes, we first found each local maximum in angular velocity, occurring at *t*_max_, and the local minima immediately before and after, at *t*_1_ and *t*_2_. If the angular change during *t*_1_ and *t*_2_ was larger than rotational diffusion, such that$\left|\Delta \theta \right|>7\sqrt{D_{r}(t_{2}-t_{1})}$, with *D_r_* = 0.1 rad^2^/*s*, we considered the trajectory to be undergoing angular change during the time interval around *t*_max_ such that the angular speed ω(*t*) satisfied the condition |ω(*t*_max_)| − |ω(*t*)| < 0.7Δω with Δω = max[|ω(*t*_max_)| − |ω(*t*_1_)|, |ω(*t*_max_)| − |ω(*t*_2_)|]. For this analysis, we chose thresholding parameters such that the detection of switch or pause was correct by visual inspection. We identified switches in direction when the angular change was greater than 90° and pauses when the angular change was less than 90°.

### Imaging rotating, tethered cells in the flow cell used to impose stepped changes in serine concentration.

A flow cell (1 mm by 4 mm by 40 mm) was used for the tethered-cell experiments, so that we could impose stepped changes in the serine concentration by controlling the flow of medium. Previous studies have used anti-flagellum antibodies to tether *E. coli* and *P. aeruginosa* bacteria to a surface (11, 15). However, we did not use antibodies because *P. aeruginosa* flagella adhered to the coverslip surface spontaneously.

One end of the flow cell was connected to both the serine-containing CB and the serine-free CB, each medium with its own tubing. A peristaltic pump (Watson Marlow, Wilmington, MA) at the other end of the flow cell could draw the solution through the chamber at a flow rate of 116 µl/s. This allowed new solution from the inlet tubing to reach the center of the flow cell in about 0.5 s and replace ~89% of the original solution in 4 s (see Fig. S5 in the supplemental material). Stepped-up and stepped-down changes in serine concentration were controlled by switching the clamps on the inlet tubes. The flow imposing a stepped change in the environment lasted for 4.5 s, and images were taken immediately after the flow stopped.

After each stepped change, we waited for at least 8 min to let the cells adapt to the current environment before imposing the next stepped-change stimulus. We imaged spinning cells in the middle of the flow cell by using a 60× oil-immersion phase-contrast objective. Images were taken at 45 frames per s for 2 to 5 min. The chamber and all solutions were maintained at 30°C.

### Analysis of the spinning of tethered cells.

The (*x*, *y*) coordinates of cell centroids were detected by using ImageJ, and the time-averaged <*x*> and <*y*> positions were defined as the coordinates of the center of rotation. For each frame, the angular position of the cell was measured by the angle of the vector (*x* − <*x*>, *y* − <*y*>). The instantaneous spinning speed between consecutive frames was derived accordingly. CW, CCW, and pause phases of the flagellum were identified by scanning a 7-frame time window across the series of instantaneous angular velocities and fitting a linear function, describing angular velocity as a function of time, over each time window. For each time window, the slope of the fitted line measured the local derivative of spinning frequency. For slopes with absolute values above a threshold, the midpoint of the associated time window was defined as the boundary of two rotational phases. The threshold was defined individually for each cell as 1/5 of the average absolute value of the slope for the steepest 0.2% of the slopes. Between two consecutive rotational-phase boundaries, we fitted a line segment to the data and used this to calculate the average spinning frequency. A spinning frequency higher than 0.5 s^−1^ was defined as CCW rotation of the cell body (CW rotation of the flagellum), a spinning speed lower than −0.5 s^−1^ was defined as CW rotation of the cell body (CCW rotation of the flagellum), and a spinning speed between −0.5 s^−1^ and 0.5 s^−1^ was defined as a pause. The values for the parameters were manually picked. Values would be adopted if the phase detection of CW and CCW was correct by visual inspection. Phase detections were done with custom-written codes in MatLab.

## SUPPLEMENTAL MATERIAL

Figure S1  (A) Microfluidic device design. For measurements of swimming trajectories, the size of the observation channel is 2,000 µm by 400 µm by 130 µm (L by W by H). For measurements of the cell density profile, the size of the observation channel is 600 µm by 200 µm by 130 µm (L by W by H). (B) Transit time measurements in a 200-µm by 200-µm field of view. The background color indicates a serine gradient along the *y* axis. Two trajectories, trajectory 1 and trajectory 2 (blue lines), each have four detected switching events (red circles). Transit time is defined as the elapsed time between two consecutive switches. For trajectory 1, transit times are the durations of the segments between 1 and 2, 2 and 3, and 3 and 4. We define the up-gradient segments as those which have an endpoint with a *y* coordinate larger than that of their start point (e.g., *y*_2_ > *y*_1_) and down-gradient segments as those which have an endpoint with a *y* coordinate smaller than that of their start point (e.g., *y*_3_ < *y*_2_). (C) Measurements of the cell density profile. A graph of cell distribution in the channel is presented. To avoid distortions caused by the boundaries, we include only those cells whose centroids fall in the rectangular region bounded by the dashed red line (388 µm by 188 µm, with margins 6 µm away from the upper, left, and right edges of the chamber). The region is subdivided along the *y* axis into 15 identical areas, and the number of cells in each area is counted and normalized to the total cell number to get the probability *P*(*y*). The probability over 300 consecutive frames is averaged in Fig. 4C and D. Download Figure S1, EPS file, 0.8 MB

Figure S2  The probability distribution of angular change during pauses and switches in a serine gradient equals 0.225 µM/µm. Red, direction switch; pink, pause. Compared with the data shown in Fig. 1D, the distribution is unaffected by the presence of a gradient. Download Figure S2, EPS file, 0.3 MB

Figure S3  Directional correlation function of nonswitching runs without pauses (A) and with pauses (B). Experiment data are fitted with the following function: <cos(φ)> = *c* exp(−2*D*_1_*t*) + (1 − *c*) exp(−2*D*_2_*t*). Rotational diffusion was obtained from the fit of this slowly decaying exponential function. Rotational diffusion equals 0.026 rad^2^/s in (A) and 0.038 rad^2^/s in (B). Download Figure S3, EPS file, 0.7 MB

Figure S4  Probability distribution of relative difference in speeds before and after a direction switch. *v*_1_ is the average running speed before a direction switch. *v*_2_ is the average running speed after the same direction switch. The symmetric distribution with a single peak at 0 indicates that the run and reverse speed are the same. Download Figure S4, EPS file, 0.3 MB

Figure S5  For tethered-cell experiments, we measured fluorescence intensity to determine the dynamics of the chemoattractant concentration for stepped-up (A) and stepped-down (B) stimulus. Solution from the inlet tubing reaches the center of the flow cell in about 0.5 s and replaces ~89% of the original solution in 4 s. Download Figure S5, EPS file, 0.5 MB

Figure S6  Schema of the chemotaxis signaling pathway. See the text for more details. Download Figure S6, EPS file, 0.6 MB

Table S1  Summary of sample sizes, instantaneous speeds, angular changes, and time percentages of pause and switch of swimming trajectories in microfluidic channels. Table S1, DOCX file, 0.01 MB

Table S2  Summary of sample sizes, rotation frequencies and durations, and percentages of pause time in tethered-cell experiments. Table S2, DOCX file, 0.01 MB

Table S3  Parameters for the simulation. Table S3, DOCX file, 0.01 MB
