# Supplementary material for: Singly Flagellated Pseudomonas aeruginosa Chemotaxes Efficiently by Unbiased Motor Regulation
Source: mBio. 2016 Apr 5;7(2):e00013-16. doi: 10.1128/mBio.00013-16 (PMC4817248; doi:10.1128/mBio.00013-16)
Supplement: Table S1 — Summary of sample sizes, instantaneous speeds, angular changes, and time percentages of pause and switch of swimming trajectories in microfluidic channels. [file mbo002162752st1.docx]

**TABLE S1** Summary of sample size, instantaneous speed, angular change and time percentage of pause and switch of swimming trajectories in microfluidic channels.

|  | # of trajectories tracked | Average duration  of a tracked trajectory (s) | # of trajectories with more than 2 switches | Instantaneous speed (µm/s)  (Mean±SD) | Relative probability | | Angular change (°) (Mean±SD) | | Percentage of total tracked time | |
| --- | --- | --- | --- | --- | --- | --- | --- | --- | --- | --- |
|  |  |  |  |  | pause | switch | pause | switch | pause | switch |
| Buffer  (no gradient) | 200 | 4.4 | 92 | 36.4 ± 17.2 | 24% | 76% | 27 ± 25 | 166 ± 15 | 4.3% | 8.8% |
| Gradient = 0.225 µM/µm | 644 | 4.4 | 235 | 40.5 ± 18.4 | 22% | 78% | 38 ± 27 | 165 ± 16 | 3.7% | 7.7% |
