# Supplementary material for: Singly Flagellated Pseudomonas aeruginosa Chemotaxes Efficiently by Unbiased Motor Regulation
Source: mBio. 2016 Apr 5;7(2):e00013-16. doi: 10.1128/mBio.00013-16 (PMC4817248; doi:10.1128/mBio.00013-16)
Supplement: Table S2 — Summary of sample sizes, rotation frequencies and durations, and percentages of pause time in tethered-cell experiments. [file mbo002162752st2.docx]

**TABLE S2** Summary of sample size, rotation frequency, duration and percentage of pause time in tethered-cell experiments.

| Serine  concentration | # of  bacteria | Rotation Frequency (s^-1^) (Mean±SD) | | | Rotation duration (s)  (Mean±SD) | | Percentage of pause time associated each combination of rotation directions* (%) (Mean±SD) | | | |
| --- | --- | --- | --- | --- | --- | --- | --- | --- | --- | --- |
|  |  | CCW | CW | CCW | | CW | CCW–CCW | CW–CW | CCW–CW | CW–CCW |
| Buffer | 46 | 2.69±1.32 | 2.55±1.19 | 1.36±1.53 | | 1.17±1.22 | 0.65±1.16 | 0.91±1.18 | 1.39±2.23 | 0.79±1.43 |
| 100 nM | 11 | 2.48±1.25 | 2.69±1.62 | 1.46±1.91 | | 1.17±1.28 | 0.42±0.38 | 1.62±2.36 | 1.17±1.60 | 1.68±2.03 |
| 1 µM | 12 | 2.74±1.22 | 2.29±1.28 | 1.32±1.50 | | 1.12±1.03 | 0.54±0.50 | 0.83±0.65 | 0.74±0.94 | 0.52±0.63 |
| 10 µM | 17 | 2.42±0.79 | 2.71±1.25 | 1.39±1.47 | | 1.21±1.29 | 0.92±2.73 | 1.10±1.58 | 0.85±0.68 | 0.66±1.81 |
| 100 µM | 17 | 2.68±1.07 | 2.91±1.37 | 1.41±1.47 | | 1.13±0.99 | 0.79±1.89 | 0.82±0.77 | 0.42±0.37 | 1.02±1.18 |
| 1 mM | 21 | 2.96±1.15 | 2.73±1.05 | 1.66±1.84 | | 1.28±1.22 | 0.34±0.42 | 0.71±0.70 | 0.91±1.43 | 0.26±0.40 |
| 10 mM | 17 | 2.36±0.82 | 2.43±1.14 | 1.56±1.90 | | 1.25±1.22 | 1.04±1.53 | 1.43±1.94 | 1.00±1.04 | 2.13±3.84 |

*CCW–CCW, CW–CW means state does not change after the pause. CCW–CW, and CW–CCW means the direction of rotation switches from CCW to CW, and CW to CCW after the pause, respectively.
