# Supplementary material for: Singly Flagellated Pseudomonas aeruginosa Chemotaxes Efficiently by Unbiased Motor Regulation
Source: mBio. 2016 Apr 5;7(2):e00013-16. doi: 10.1128/mBio.00013-16 (PMC4817248; doi:10.1128/mBio.00013-16)
Supplement: Table S3 — Parameters for the simulation. [file mbo002162752st3.docx]

**TABLE S3** Parameters for the simulation.

| Regulation of chemoreceptor activity* | | | | | | | | | | | | | | Regulation of motor switching† | | | | |
| --- | --- | --- | --- | --- | --- | --- | --- | --- | --- | --- | --- | --- | --- | --- | --- | --- | --- | --- |
| N | α | m_0_ | a_0_ | K_A_ (µM) | K_I_ (µM) | K_R_ | K_B_ | a_R_ | a_B_ | r_R_ | r_B_ | V_R_(0) (s^-1^) | V_B_(0) (s^-1^) | lnA-k_1_ | lnA-k_2_ | β_1_ | β _2_ | γ |
| 6 | 1.7 | 1 | 0.5 | 1000 | 1 | 0.5 | 0.5 | 0.1 | 0.75 | 4 | 4 | 0.05 | 0.05 | -3.2 | -3.2 | 6.0 | 6.0 | 0.5 |

* K_I_ is based on earlier microcalorimetric titration analysis in *P. aeruginona* (M. Rico-Jiménez, F. Muñoz-Martínez, C. García-Fontana, M. Fernandez, B. Morel, A. Ortega, J.L. Ramos, T. Krell. Mol. Microbiol. 88(6):1230–1243, 2013, doi: 10.1111/mmi.12255). K_A_ is estimated from the response curve in Fig. 3AD. Adaptation rates V_R_(0) and V_B_(0) are estimated from Fig. 3F. For simplicity, we let V_R_(0) = V_B_(0)

†The parameters in motor switching regulation, namely ln A-k_1_, ln A-k_2_, β_1_, β_2_, and γ, are ﬁtted using the tethered-cell experimental data Fig. 3AD. All other parameters (N, α, m_0_, a_0_, a_R_, a_B_, r_R_, and r_B_) are adopted from previous studies (L. Jiang, Q. Ouyang, Y. Tu. PLoS Comput. Biol. 6(4):e1000735, 2010, doi: 10.1371/journal.pcbi.1000735; T.S. Shimizu, Y. Tu, H.C. Berg. Mol. Syst. Biol. 6:382, 2010, doi: 10.1038/msb.2010.37.)
